# Olfactory Cues in the Odour Plume of Predatory Fish Reduce Foraging and Elicit Anti-Predator Behaviour in the European Green Crab *Carcinus maenas*

**DOI:** 10.3390/ani16050828

**Published:** 2026-03-06

**Authors:** Jonathan W. Burnett, Hannah Ohnstad, Amber M. Jones, Jörg D. Hardege, Helga D. Bartels-Hardege

**Affiliations:** 1 School of Natural Sciences, Biological and Environmental Sciences, The University of Hull, Hull HU6 7RX, UK; 2 National Oceanography Centre, School of Ocean and Earth Science, University of Southampton, Southampton SO16 6YD, UK; amber.jones@soton.ac.uk

**Keywords:** European green crab, *Carcinus maenas*, predator recognition, invasive species, anti-predator behaviour, chemical deterrent, integrated pest management

## Abstract

The European green crab (*Carcinus maenas*) is an intertidal crustacean that has extended its invasive range globally and is regarded as a major pest species for bivalves, impacting coastal food chains and aquacultures. Crabs primarily use chemosensory cues to sense their environment for feeding to avoid predation and to locate mates and induce mating. Consequently, known attractants including food baits are frequently employed in trapping and control efforts. Here, we investigate the effects of introducing a predatory fish odour to the environment and show that it elicits anti-predator behaviour in *C. maenas*. A 45% reduction in crab foraging and feeding behaviour was observed when predator odour was introduced compared with food control experiments. A further 23% reduction (68% reduction overall) in feeding behaviour was observed after crabs were exposed to (though housed separately from) the same seawater as the live predator for several days. This highlights the increased awareness or continuous stress of these habituated crabs towards predator odour. This study also presents potential for application as a deterrent in integrated pest management strategies for this global invader.

## Simple Summary

The interaction between a predator and its prey is a race for survival, characterised by both sides employing specific tactics to increase their chances. Amongst the key mechanisms enabling prey and predator to detect each other is the sense of smell. Odour cues often alert either side to the presence of the other, and prey typically employ ways to hide or mask their presence, as famously observed in deer, where fawn have no scent at all. Detecting a predator by its smell should initiate hiding or flight responses in marine animals such as the European green crab, *Carcinus maenas*. However, the question remains whether animals living permanently in the odour plume of a predator can still detect this or whether they adapt to the smell when no physical harm occurs. This type of acclimation or learning would benefit ambush hunters such as predatory fish as prey would become desensitised. Here we tested if such desensitisation occurs in green crabs when exposed to sea bream odour and found that the opposite is the case: crabs become hypersensitized and react stronger to predator odour over time, decreasing foraging for food and initiating hiding or escape behaviour. As many crab species are globally invasive, the fact that they increasingly avoid predator odour even when no physical threat is involved can provide a starting point to explore the potential of odour as a deterrent against crabs, with implications for aquaculture and fisheries once the chemical nature of the active compounds has been elucidated.

## 1. Introduction

Predator–prey relations can usually be defined as the interactions between two organisms where one is hunted for consumption by the other. Mediated by habitat characteristics and the physical–chemical environment [1], these interactions influence a range of parameters in aquatic communities, including biodiversity, water quality and mediation of trophic cascades. Often, predators directly regulate the recruitment and survival of prey species and indirectly regulate factors such as the size, structure, growth, habitat choice, foraging and behaviour of prey populations [1,2]. Almost all aquatic species are subject to predation during their life [3], and with many organisms occupying environments shared with predators (as well as competitors), foraging for resources increases vulnerability to attack [4]. To minimise this risk, animals employ behavioural strategies to ensure survival. The effective response to and avoidance of predation require identification of potential predators via sensory recognition to initiate anti-predator behaviour [5,6]. Cues can be detected using a variety of sensory modes, including auditory, visual, olfactory, social, and tactile sense mechanisms [7]. Potential dangers such as sudden movements, shadows from looming objects, or species-specific cues, including visual recognition and scent, allow prey to identify or distinguish predators from non-predatory species and make the appropriate reactive responses.

Prey species deploy a diverse range of preventive strategies to minimise encounters with potential threats. Camouflage [8] has been observed in a variety of species such as the cuttlefish *Sepiida* sp. [9], the Peacock mantis shrimp *Odontodactylus scyllarus* [10], and the Decorator crab *Majoidea* sp., which achieve background matching utilising materials such as algae, attaching them to its carapace [11]. The European green crab *Carcinus maenas*, when placed on a variety of backgrounds of different colours and luminance, acclimates within 1 h [8]. This suggests that camouflage in *C. maenas* is precautionary rather than a rapid behavioural response to a detected predator. *C. maenas* tends to remain inactive and rely on this protective cryptic colouration to reduce visibility and to maximise detection difficulty for many predators [12,13]. This strategy may be effective for avoiding vertebrate predators that rely on visual cues when hunting species such as most fish and seabirds. It is currently not established though whether hiding is effective against predators utilising chemosensory senses.

Prey species may also increase their chances of survival by selecting habitats that are less frequented by predators and/or provide cover [14]. For example, the European lobster *Homarus gammarus* protects itself from risk of attack by inhabiting rocky crevices [15], and *Carcinus maenas* often select eelgrass beds to escape fish [16]. When faced with high predation pressures, prey’s habitat choice is an integral factor ensuring successful reproduction [17]. Increased nocturnal activity levels in prey species reduce diurnal predator exposure. By foraging during periods when known predators are less active, prey species, including the spiny lobster *Panulirus argus* [18], can effectively exploit resources in their respective habitats [14]. Prey may also reduce foraging time upon detecting a predator as an avoidance strategy [19], as observed in the mud crab *Panopeus herbstii*, which decreases its foraging activity in the presence of chemical cues from its primary predator, the blue crab *Callinectes sapidus* [20].

Whilst behavioural adaptation is a critical aspect of survival in the marine environment, periods of restricted foraging potentially reduce prey growth and fecundity [21], leading species to mitigate this cost either by evaluating local level of risk to establish the appropriate time to initiate anti-predator tactics or by only responding to primary cues. The latter comes with its own risks; prey may not always detect the signals/scent of a stealthy or fast predator in adequate time to avoid attack/consumption [4,18], demonstrating that whilst avoidance clearly benefits survival, these strategies alone cannot guarantee that predators will not be encountered.

When detecting a predator, the predator’s chemical signature is often the most reliable indication of a threat, and a variety of defensive behaviours may then be utilised by prey species. In crustaceans this includes burrowing and hiding in sand or crevices to seek refuge. This is a tactic used by many prey species in avoiding detection [22]. Prey can use chemical cues to assess threats quantitively via odour cue concentration, or qualitatively via differences in cues based on predator diet, to differentiate predator size and biomass. They may exhibit heightened avoidance behaviours if a predator has recently consumed conspecifics, indicating a direct risk of predation [23]. The mud crab *Panopeus herbstii*, when presented with a variety of predators (blue crab, *Callinectus sapidus*), diets and biomass treatments, exhibits hiding behaviour as well as reduced foraging and feeding, though only when presented with cues from large *C. sapidus* individuals. The presence of multiple small *C. sapidus* resulted in similar decreased foraging, as seen with large individuals, suggesting that *P. herbstii’s* perception of predator size is directly related to biomass. Heightened anti-predator responses were observed when presented with *C. sapidus* treatments fed on *P. herbstii*, compared to those that were fed on oyster diets or presented with crushed conspecifics. This demonstrates qualitative cues’ influence on intraspecific threat perception, as well as indirect interactions [23].

Whilst prey’s recognition and response to predatory threats are critical to the survival of prey populations [24,25], prey failing to respond adequately are subject to a phenomenon known as enhanced predation—more commonly referred to as prey naiveté [26]. Species that lack exposure to specific predators, due to either not co-occurring over a geographical range or lacking evolutionary history, will fail to recognise them as a threat and thus do not exhibit the appropriate or sufficient anti-predator responses [27,28]. The introduction of an invasive predator to an ecosystem disrupts predator–prey dynamics, often leading to higher predation rates of native prey populations [29,30]. Significant population declines in native bivalves in costal ecosystems across the invasive range of the European green crab, *Carcinus maenas* [31], and the blue crab, *Callinectes sapidus*, in the mediterranean [32] are prime examples.

There is however conflicting evidence suggesting that some prey have an innate capacity to identify predatory threats that they have never previously encountered; these are often, but not always, prey that share an evolutionary history with a specific predator [27]. Associative learning, an organism’s ability to link stimuli to a specific outcome, allows prey to adapt or modify their behaviour based upon prior experience to minimise predation risk [33]. By utilising such learning mechanics, prey can enhance their chances of survival via improved recognition to threats [34]. Independent of whether a response to predator cues is innate or learned, very little is known about potential changes in the response towards predator odour over time [35]. Constant exposure to predator odour can induce two significantly different outcomes, habituation or sensitisation, with the literature showing no clear indication which pathway is dominant [36]. There are examples of prey over time failing to detect the predator odour as it blends in as background noise; alternatively, continuous exposure to predator cues can induce chronic stress, increasing sensitivity towards predator odour [37].

In this study we examine the behavioural responses of the European green crab, *C. maenas*, to the odour of Gilt-head sea bream *Sparus aurata*, a known predator of this crab species. *C. maenas* is a decapod crustacean ranking among the top 100 most invasive species in the world [38]. It is omnivorous, with a high tolerance and adaptability to pH, temperature and salinity that can be attributed to natural tide cycle exposure in the intertidal zones across its native and invasive ranges. *C. maenas* exhibits several common anti-predator behaviours, including shelter use, i.e., hiding in crevices, under rocks or burying into sand; aggressive displays such as spreading and waving claws to intimidate predators; and rapid escape movements to flee from threats [38]. Some behavioural responses, e.g., response to prey cues in a lowered pH, have been observed to be sex-specific [39].

Our initial hypothesis is that exposure to the odour of *S. aurata* elicits anti-predator behaviour and disrupts feeding in *C. maenas*. We then test the hypothesis that *C. maenas* becomes desensitised (habituates) when exposed to predator odour over a period of time without experiencing any consequences or negative associations.

## 2. Materials and Methods

### 2.1. Specimens for Study

*C. maenas* (*n* = 220) were hand collected at low tide by local fishermen from the coast of Faro (36°59′46.4″ N, 7°58′57.0″ W), Portugal, and delivered to the Ramalhete field station located in the Parque Nacional da Ria Formosa, an intertidal but full-strength seawater lagoon (36 ppm ± 0.5) near Faro, during September 2022. They were measured for carapace width using a calliper and stored (*n* = 60 per tank) in four large 1500 L tanks (W = 1.5 m, L = 1.5 m, H = 80 cm) containing hollow clay bricks and 20 cm plastic tubing (7.5.cm diameter) to use as hides. The tanks were supplied with flow-through, sand-filtered natural seawater pumped from the Ramalhete estuary (pH 8.2–8.3, 21–25 °C) and seawater parameters were controlled as described by Sordo et al. 2016 [40]. Black lids were placed over the tanks to reduce visual stressors and allow for the control of photoperiod to match autumn conditions in Faro. Males and females were stored together to allow for natural behaviours such as pair mating.

The crab sample group was split into two: 60 housed in holding tanks with no other species representing the control group, and 60 housed in tanks with incoming water flow from the outlet pipe of *Sparus aurata* holding tanks, ensuring crabs could smell live predators in the environment without any physical contact. Both sets of crabs were allowed to habituate in holding tanks for 1 week. Crabs were fed every 4 days on a diet of frozen cooked mussel meat (*Mytilus edulis*) and were deprived of food three days prior to experiments to standardise hunger. All individuals were measured using carapace width (cm) as a measure of size and marked for identification for the duration of the study using a general use oil-based Dykem marker pen suitable for underwater use, non-toxic for use on crustaceans.

### 2.2. Cues and Conditions Tested

Feeding Cue: Raw unsmoked bacon meat was chosen as a suitable feeding stimulant. Although not a natural food source for the crabs, it has been used successfully on *C. maenas* [41] as it is very reliable for catching crabs. Bacon was blended and filtered to ensure only its juices mixed into a proprietary slow-release semio-chemical delivery matrix of carboxy-cellulose (Sigma-Aldrich, Gillingham, UK) [42] to ensure the dispersal rate matched that of the conditioned water. ‘Conditioned water’ is the term used to describe the sea bream water used as the predator cue; this water was collected from 3000 L holding tanks containing 30 live *S. aurata* and used as the predator odour. Conditioned water was mixed into the same delivery matrix as the feeding stimulant. The conditions tested were:

Control vs Food (CvF)—feeding stimulant against blank matrix, using crabs habituated to an environment without the predator. Sea Bream + Food vs. Control (SB + FvC)—feeding stimulant with predator odour against blank matrix, using crabs habituated to an environment without the predator. Sea Bream (Habituated) + Food vs. Control (SBH + FvC)—feeding stimulant with predator odour against blank matrix, using crabs acclimated to/living in the wake of the predator.

### 2.3. Y-Shaped Olfactometer and Experimental Design

Two Y-shaped olfactometer tanks were used in tandem in experiments as described by Ohnstad et al. 2024 [43] to provide a standard method for assessing animal behaviours [44]. Each condition was tested in natural seawater pumped directly into the olfactometers from the local estuary (pH 8.2–8.3), which entered each tank at the two inlet points of the ‘Y’ and left via the base [43]. Flow rate was set at 1 L min^−1^ (500 mL min^−1^ per arm), ensuring equal diffusion of chemical cues and removal of residual odours from prior runs. Cue dispersal rate was assessed previously [43], establishing that odours took approximately 5 min to completely diffuse to the opposite side of the olfactometer. Olfactometers were filled with seawater to 12 cm depth, and the base of the tanks was covered in a layer of washed marine sediment (2.5 cm thick). After measuring pH, temperature, and salinity, the matrices were placed into stainless-steel tea strainers to weigh them down and placed at the tips of the Y-shaped olfactometers, allowing diffusion into the water flow. The cue sides were switched at random intervals to eliminate any tank preference (see [43] for details). Baseline responsiveness to food stimuli was assessed by positive control (CvF), exposing *Carcinus maenas* deprived of food for three days prior and observing their behaviour. For each test, a single crab was held in a plastic container at the base of the olfactometer and given two minutes to settle. Cues were placed at each side of the Y-tips of the olfactometers. *C. maenas* were released after acclimation and observed for 5 min [43]. Behaviours recorded were initial reaction time (in seconds), marked by the start of rapid antennular flicking or movement; cue choice (e.g., cue/control/neither); behavioural response (e.g., flicking, active, grab, bury, escape); and time taken to reach the cue (in seconds). If a crab did not respond or make a choice within the 5 min period, the trial was excluded from location time analysis but retained for the initial reaction time analysis [43]. This was because, even in cases where they did not reach a cue, all crabs displayed behavioural changes such as increased antenna flicking (detecting a cue), burial (escape behaviour), wafting (moving chelipeds to create current) or other physical responses (i.e., raised chelipeds as defence posture).

For each condition, 40 repeats were carried out. Crabs were chosen randomly from the sample tanks daily, alternating which holding tank the individuals were selected from to ensure individuals were allowed at least four days respite between experiments to minimise handling stress and potential effects of short-term memory. Neither weight or sex distribution differed significantly between the groups, as animals were picked at random but selected for sex (see Supplementary Figures S1 and S2). To reduce bias for physiology, all crabs were adults with a size large enough to potentially reproduce (>3 cm carapace diameter) [45].

### 2.4. Statistical Analysis

All analyses were conducted using R studio v4.4.1 [46]. All data samples were tested for normal distribution with the Shapiro–Wilk test [47] prior to analysis. As the data was not normally distributed (*p* ≤ 0.05) and was discontinuous, non-parametric tests were chosen. For both the feeding/foraging experiments and the observed behaviour experiments, treatment effects on categorical decision outcomes (Control, Food, No Decision) across the three conditions (CvF, SB + FvC, SBH + FvC) were analysed using multinomial logistic regression. It should be clarified that ‘No decision’ at the time of carrying out bioassay experiments refers to no choice to move to either cue presented/arm of the olfactometer being made, where crabs remained stationary. However, this should still be considered a choice/decision itself, given that remaining still and relying on not being seen is a known anti-predator response, and behavioural differences can be quantified against control experiments. Model fit was evaluated with likelihood ratio tests comparing the full model (including treatment condition) against a null model without predictors. To further explore associations between treatment groups and decision categories, Multiple Correspondence Analysis (MCA) was performed [48].

In the case of continuous variables, ‘Initial Reaction’ and ‘Time to Cue’ responses, both the assumptions of normality and homogeneity of variances were violated following Shapiro–Wilk and Levene’s tests. Therefore, linear models were fitted with the treatment condition as a fixed factor. Model fit was evaluated using F-tests, and post hoc pairwise comparisons between treatment groups were conducted using estimated marginal means (emmeans). Data visualisation was performed in R using the *ggplot2* package, including bar plots for categorical outcomes, boxplots for continuous measures, and MCA biplots (*factoextra* package) for multivariate representations. For analysis of size and sex, a generalised least squares (GLS) model was implemented using the nlme package (v3.1-162, [49]). Model comparisons and likelihood ratio tests were performed with functions from nlme, and *p*-values for GLS models were derived from likelihood ratio tests. Graphical assessment of model assumptions was conducted through residual plots and Q-Q plots, generated using the ggplot2 package (v3.4.3 [50]), allowing visual inspection of homoscedasticity and normality of residuals

## 3. Results

### Feeding and Foraging Activity

**Figure 1:**
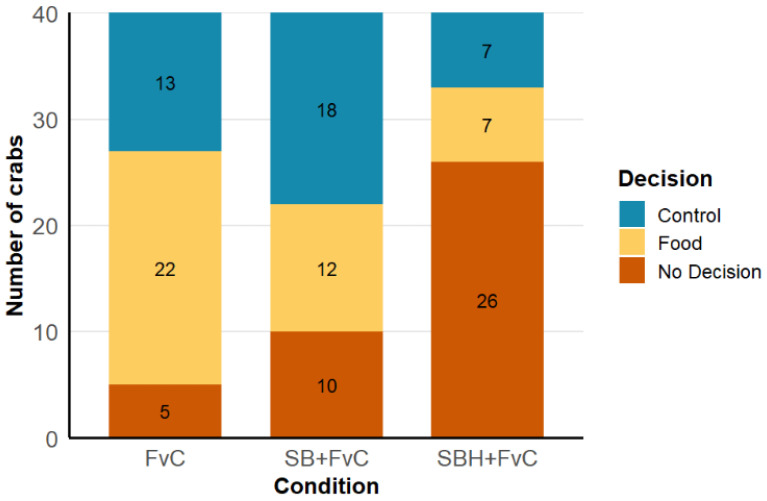
Total number of individual crab decisions (sample *n* = 40) in each of the three experimental conditions (Control= Blue, Food= yellow, No Decision= orange). Experimental groups tested were FvC (Food vs. Control), SB + FvC (Sea Bream + Food vs. Control), and SBH + FvC (Sea Bream Habituated + Food vs. Control). Figures in the bars represent no. of crabs for each decision. No decision describes crabs that remain stationary with no movement towards either arm of the olfactometer (see Section 2).

As shown in Figure 1 the number of crabs choosing the food cue reduced in the presence of predator odour [FvC: (*n* = 22), SB + FvC: (*n* = 12)], representing an observed 46% reduction in foraging behaviour. This number decreased even further in animals that had been constantly exposed to the scent of *Sparus aurata* [SBH + FvC: (*n* = 7)], representing a further 23% reduction and so a total reduction in foraging by 68% overall when compared with CvF. Likelihood ratio tests comparing a multinomial logistic regression model, including treatment to a null model without predictors, revealed a significant effect of treatment on crab decision-making (χ^2^ = 30.62, df = 4, *p* = 3.65 × 10^−6^). Crabs exposed to combined sea bream and food odours (SB + FvC) showed a trend toward reduced selection of the food cue compared to controls (β = –0.93, *p* = 0.068), while habituated crabs (SBH + FvC) exhibited a similar nonsignificant decrease (β = –0.53, *p* = 0.41). Notably, habituated crabs (SBH + FvC) were more likely to make no decision to run into either arm of the olfactometer relative to FvC (β = 2.27, *p* = 0.0008), suggesting increased hesitation following prolonged predator odour exposure (Supplementary Analysis 3).

**Figure 2:**
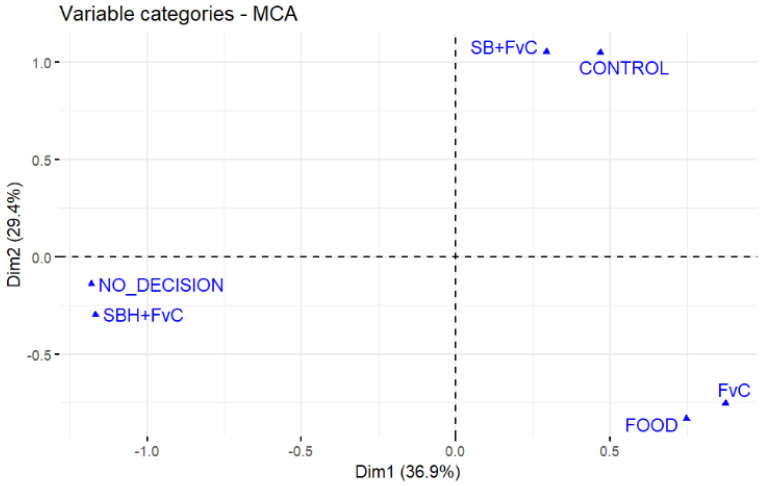
Multiple Correspondence Analysis (MCA) plot showing the associations between decision outcomes and treatment conditions. Points represent category positions along the first two MCA dimensions (Dim1 = 36.9%, Dim2 = 29.4%), which together explain 66.3% of the total variance. Treatment groups FvC (Food vs. Control), SB + FvC (Sea Bream + Food vs. Control), and SBH + FvC (Sea Bream Habituated + Food vs. Control) and decision categories (Food, Control, No Decision) are plotted to visualise relationships.

To identify the behavioural outcomes most strongly associated with each treatment, Multiple Correspondence Analysis (MCA) was performed, revealing distinct separation between behavioural outcomes and treatment groups. The FvC treatment showed a stronger association with choosing food, while the SB + FvC treatment was more associated with choosing the control. In contrast, the SBH + FvC group was closely linked to the ‘no decision’ outcome, indicating increased indecision following prolonged predator odour exposure (Figure 2; Supplementary Figures S3 and S4).

**Figure 3:**
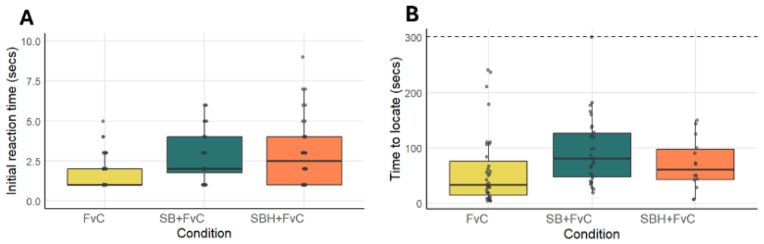
Individual crabs’ initial reaction time (**A**) and time to locate the cue (**B**) in seconds, across three experimental conditions: FvC (Control vs. Food; blue), SB + FvC (Sea Bream + Food vs. Control; grey), and SBH + FvC (Sea Bream habituated/acclimated crabs + Food vs. Control; red). Panel A includes all individuals (*n* = 40). Panel B includes only crabs that made a decision, with sample sizes as follows: FvC (*n* = 35), SB + FvC (*n* = 30), and SBH + FvC (*n* = 14). Jittered points overlay the boxplots to show individual variation. A dashed baseline at y = 300 s is included in panel B to show maximum experiment time. Error bars represent standard error of the mean, with the line inside each boxplot indicating the median time in seconds.

The treatment condition did not have an effect on initial reaction time (linear model: F(2, 76) = 1.78, *p* = 0.176). Although crabs in the SB + FvC group tended to exhibit longer initial reaction times compared to controls (estimate = 2.995 s, *p* = 0.063), this difference was not statistically significant. No significant difference was observed between the SBH + FvC and control groups. Treatment condition did not have an effect on the time taken by crabs (that made a decision) to locate the cue (linear model: F(2, 76) = 2.60, *p* = 0.081). However, pairwise comparisons revealed that crabs exposed to the SB + FvC treatment required significantly more time to locate the cue compared to controls (estimate = 34.51 s, *p* = 0.027), whereas no significant difference was observed between the SBH + FvC and control groups (Figure 3B). Neither sex nor size, across treatments, significantly influenced the time to locate the cue (Supplementary Analysis 5). The minimal adequate model was an intercept-only model, which estimated a baseline mean time of 74.71 s (SE = 7.07, t = 10.57, *p* < 0.001).

**Figure 4:**
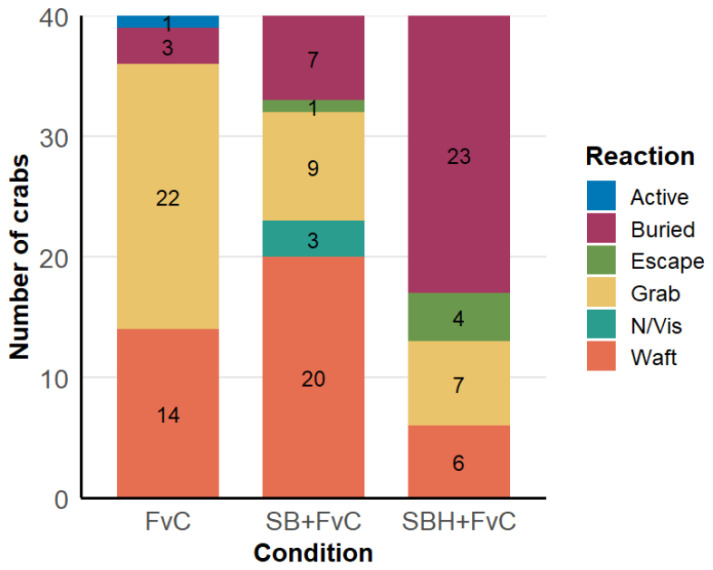
Total number of individual crab behaviours observed (sample n = 40) in each of the three experimental conditions. Experimental groups tested were FvC (Food vs. Control), SB + FvC (Sea Bream + Food vs. Control), and SBH + FvC (Sea Bream (Habituated) + Food vs. Control). N/VIS in the behaviour key = no visible reaction, crab stationary. Figures in the bars represent no. of crabs that displayed a particular reaction/behaviour.

A likelihood ratio test confirmed a significant effect of condition on crab behavioural reactions (χ^2^ = 52.42, df = 10, *p* = 9.55 × 10^−8^; Supplementary Analysis 6). Introduction of predator odour (SB + FvC) increased anti-predator responses, with buried (*n* = 7) and escape (*n* = 1) behaviours observed. Predator odour-habituated crabs (SBH + FvC) exhibited a further 60% increase in anti-predator behaviours (buried (*n* = 23) and escape (*n* = 4)) (Figure 4). Pairwise contrasts from the multinomial model support these differences: burial behaviour was higher in SBH + FvC than FvC (estimate = −0.50, SE = 0.089, t = −5.65, *p* < 0.001) and SB + FvC (estimate = −0.40, SE = 0.099, t = −4.06, *p* = 0.003), grab responses were increased in SBH + FvC versus FvC (estimate = 0.38, SE = 0.10, t = 3.79, *p* = 0.005), and wafting behaviour was higher in SBH + FvC versus SB + FvC (estimate = 0.35, SE = 0.097, t = 3.60, *p* = 0.007) (Supplementary Analysis 6, Supplementary Table S6). No significant differences were observed for active, escape, or N/Vis behaviours. These results demonstrate that predator odour and habituation enhance specific anti-predator behaviours such as burying in crabs whilst reducing the occurrence of positive behaviours such as grabbing (of food).

**Figure 5:**
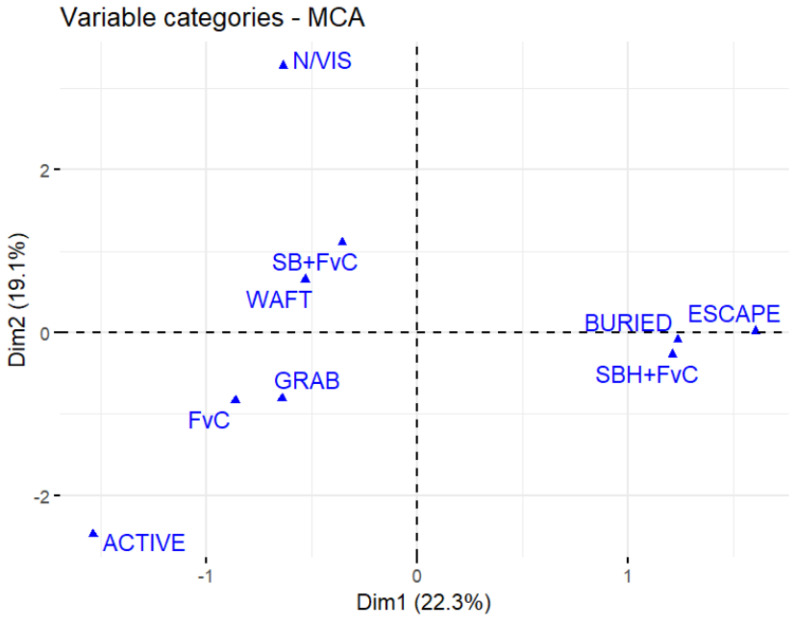
Multiple Correspondence Analysis (MCA) plot showing the associations between behaviour exhibited and condition. Points represent category positions along the first two MCA dimensions (Dim1 = 22.3%, Dim2 = 19.1%), which together explain 41.4% of the total variance. Treatment groups (FvC, SB + FvC and SBH + FvC) and decision categories (Waft, Grab, Active, N/vis, Escape and Buried) are plotted to visualise relationships.

To identify the behavioural outcomes most strongly associated with each treatment, Multiple Correspondence Analysis (MCA) revealed distinct separation between the response outcomes and treatment groups. The FvC treatment showed a stronger association with grabbing and active behaviour, while the SB + FvC treatment was more linked to wafting and FvC. In contrast, the SBH + FvC group was closely linked to escape and burial behaviours, indicating increased avoidance/hiding behaviour following prolonged predator odour exposure (Figure 5; Supplementary Figures S5 and S6).

## 4. Discussion

This study shows that, as hypothesised, upon detecting the odour of a predator, in this case the Gilt-head sea bream *Sparus aurata*, *C. maenas* either take longer to forage for food or abandon foraging altogether to initialise escape behaviours. This effect is increased when *C. maenas* continually detects the odour of the predator over an extended period, highlighting that in contrast to our hypothesis, crabs do not habituate to predator odour. Here we provide clear evidence that the introduction of *S. aurata* odour alone without any direct physical contact elicits anti-predator behaviour and disrupts feeding in *C. maenas* (Figure 1). The slight increase in initial reaction time and significant increase in time to forage to the cue location can be attributed to *C. maenas*’s recognition of the odour as a potential threat, triggering survival mechanisms to reduce visibility and thus accounting for the reduction in foraging and feeding behaviour [51]. Heightened vigilance is key to enable quick escape reactions to avoid predation, and this behaviour is widely observed in crabs [2].

*C. maenas* is a highly adaptable crustacean, and its memory and learning capabilities are well documented in the literature. This includes social learning and memory retention during agonistic encounters, where *C. maenas* can remember past opponents and adjust their behaviour in subsequent encounters [52,53]. C. *maenas* can recall successful routes over extended periods, suggesting a retention of long-term memory [54]. This is an advantageous ability in their intertidal and estuarine habitats when locating shelters and escape routes or remembering food sources [55]. Negative association recognition also plays a crucial role in *C. maenas*’s learning abilities. Stimuli such as electric shocks and aversive chemicals become associated with cues to avoid certain areas or behaviours in the future [56], and this behaviour extends to predator recognition, with *C. maenas* expected to remember the scent of a predator after an encounter [57]. Given this capacity for negative association and long-term memory [54], it is reasonable to propose that it would be beneficial for *C. maenas* to use this retained information to behave proactively rather than reactively to detected cues over extended periods, thus increasing its chances of survival. This should particularly be the case when continually exposed to diverse and fluctuating predator populations.

In this study, crabs had been in captivity for one week prior to experiments and thus had no predator encounters for the duration of that period, though it is unknown if individuals had experienced previous encounters with *S. aurata* in the wild. Whilst our study hypothesised that *C. maenas* individuals that were continually exposed to live *Sparus aurata* over the same period may become desensitised by the absence of negative effect association, this was not the case. Prolonged exposure to predator odour alone prior to experiments heightened anti-predator responses in experiments (Figure 4). This could be a result of constant stress or increased sensitivity to the olfactory cues. Magurran (1960) [58] reported similar findings in fish, where during extended exposure to a threat, animals become ‘threat-conditioned’ to remain alert to optimise survival. An interesting concept to explore would be whether *S. aurata* is intentionally or indirectly alerting prey to its presence to increase hunting success via manipulation of prey behaviour. This may sound counterproductive to the predator, and is less common than stealth or ambush tactics, but there are examples of predators using prey species’ own anti-predator responses against them documented in the literature. Cetaceans, including bottlenose dolphins *Tursiops truncatus* and killer whales *Orcinus orca* populations, have been observed to use splashing/bubbles and loud vocals to hunt fish [59]. The startled fish school together tightly, a behavioural response intended to reduce predation risk to individuals but that now instead makes the fish more vulnerable to co-ordinated attacks. There are also examples of predators exploiting prey’s chemo-senses, including benthic predators such as the catshark *Galeus melastomus* that excrete chemical cues that trick prey species into fleeing to the refuge of confined spaces where they become easier to catch [60]. Establishing whether this is an overlooked concept of a hunter minimising effort via prey manipulation would aid in better understanding the complex predator–prey dynamics between these two species. Our study also demonstrated that size and sex did not impact individual responses to a threat (Figure 3). Behavioural differences between the sexes have previously been observed in *C. maenas*’s responses to sex pheromones when pH is changed [43]. The lack of differences between the sexes highlights that sex-specific evolutionary pressures and ecological strategies do not affect detection of predators whilst they significantly impact mating. When considering the above reviewed literature, the behavioural responses of the mud crab *Panopeus herbstii* towards *C. sapidus* cues suggested that *P. herbstii’s* perception of predator size is directly related to biomass [61], with cues from multiple small crabs eliciting the same anti-predator responses to those of larger crabs [43]. As the predator cue in the current study was taken from a tank containing multiple *S. aurata*, further investigation is required to examine if similar correlations to biomass of the predator exist in *C. maenas*.

Crabs and other decapods can lose long-term memory and thus forget threat-associated cues over time due to lack of exposure [62]. *C. maenas* has been observed to display weakened avoidance behaviours to instinctive factors such as light without reinforcement of danger, demonstrating rapid learning abilities [63]. The longevity of *C. maenas* memory to predator cues, as well as whether there is a time threshold to ‘lowering its guard’, could be explored in further research. There is also the question of whether the behavioural response to *Sparus aurata* is learned or innate behaviour. A study on the isopod *Idoltea balthica*, comparing anti-predator responses to water-born cues emitted by a native predatory fish *Perca fluviatilis* to those cues that stem from an invasive crab *Rhithopanopeus harrissi*, with which the isopod shared no co-evolutionary history, revealed that *I. balthica* reduced activity only when exposed to the fish. However, the crab cue induced no anti-predator behaviour, accounting for high mortality of the isopod during exposure to the invader [28]. It is possible that different *C. maenas* populations may respond differently to those caught off the coast of Portugal, since these crabs are widespread in areas across their native and invasive range where *Sparus aurata* do not occur, presenting future studies with the opportunity to test this cue on naïve populations to evaluate whether the recognition of the predator odour is indeed learned via prior encounters or is instead an innate/evolved behavioural response shared by all *C. maenas* to this particular cue.

Roggatz et al. (2016) [64] demonstrated that the chemical cues used by *C. maenas* to control egg ventilation and larval release can be pH-sensitive and bioactivity is reduced in lowered pH conditions. Similarly, Richardson et al. (2021) [42] showed that feeding behaviour in *C. maenas* decreased under simulated ocean acidification pH conditions. Nevertheless, the responses to predator odour, in this case cuttlefish *Sepia officinalis*, were not impacted. This type of pH stability is assumed to be based on its key role to ensure survival and is similar to the lack of habituation in our current study. Future studies could examine how the intricate balance between *C. maenas* and *S. aurata* is impacted by global change, especially altered oceanic pH.

Crab invasions are on the rise, from the continuing global expansion of *Carcinus maenas* to the more recent invasions of the Mediterranean by the blue crab, *Callinectus sapidus*, which is exponentially extending its range across Europe and surrounding waters [65]. Control of invasive crab populations is unlikely due to high reproduction rates, adaptability, and widespread dispersal. Traditional control methods such as trapping or manual removal have proven ineffective, with limited success, and are labour-intensive, expensive and insufficient for large-scale management [66]. Using traditional fish baits in traps presents its own challenges, due to the high costs of baits; lack of species specificity, resulting in unwanted bycatch; and biofouling, highlighting an urgent need for alternatives. Therefore, tailored integrated pest management (IPM) strategies utilising odour cues such as pheromones or deterrents [22] to manipulate crab behaviour are urgently needed, offering a more effective and sustainable solution.

To move towards application of the sea bream cue(s) in pest management, in addition to future work aiming to understand the ecological impacts of this cue, it will be key to evaluate the chemical nature of the biologically active compound(s). Purification and identification of such deterrents have rarely been achieved [67]. Examples include Homarine and Trigonelline, which were found in blue crab urine and induce fear in mud crabs [67,68]. These two metabolites, although structurally similar, have very distinct and different biosynthetic origin pathways [67], highlighting that until the chemical nature of the sea bream cue is elucidated, its physiology remains unknown. Bioassay-guided purification [69] and advanced analytical tools, including metabolomic NMR, could be utilised for this purpose in future studies.

This research takes the first steps in highlighting a cue which deters feeding and promotes avoidance behaviour in this voracious invader, potentially laying the foundation for future research into identifying a targeted, sustainable, and ecologically sound control method for this growing international problem.

## 5. Conclusions

Whilst we still have major gaps in our understanding of the chemical odourscape of the marine environment, increasing evidence exists that odour cues govern most interactions between marine organisms. Here we show that in predator–prey interactions, one cannot always assume that habituation to predator odour leads to desensitisation of the prey and thus benefits the predator. In the case of *C. maenas*, continued exposure to the odour of sea bream *S. aurata* leads to sensitisation, significantly reducing foraging and feeding behaviour and instead *C. maenas* opts to hide (bury) or escape. It remains to be evaluated if this represents a largely overlooked trait of ambush hunters, reducing the effort required to locate prey, or if it leads to spatial segregation. Our data highlights the complexity and situation dependency of predator–prey dynamics between species.

## Data Availability

The original contributions presented in this study are included in the article/Supplementary Materials. Further inquiries can be directed to the corresponding author.

## Supplementary Materials

The following supporting information can be downloaded at: https://www.mdpi.com/article/10.3390/ani16050828/s1, Figure S1: Impact of carapace width upon crab’s responses to olfactory cues; Figure S2: Impact of crab’s sex upon responses to olfactory cues; Figure S3: Multiple correspondence analysis (MCA) of variables impacting crab behaviour (Dimension 1); Figure S4: Multiple correspondence analysis (MCA) of variables impacting crab behaviour (Dimension 2); Figure S5: Multiple correspondence analysis (MCA) of variables impacting crab behaviour (Dimension 1) based on nominal input; Figure S6: Multiple correspondence analysis (MCA) of variables impacting crab behaviour (Dimension 2) based on nominal input; Table S1: Tukey HSD post hoc comparisons of impact of carapace width upon crab behaviour; Table S2: Multinomial Logistic Regression Coefficients for Decision Making by Treatment Condition; Table S3: Linear regression model summary table examining the effect of treatment condition on time to locate the cue in shore crabs; Table S4: Linear model summary table of linear regression model examining the effect of treatment condition initial reaction time (IR) in shore crabs; Table S5: Pairwise Comparisons of Crab Behavioral Reactions by Treatment Condition.
