# Supplementary material for: Olfactory Cues in the Odour Plume of Predatory Fish Reduce Foraging and Elicit Anti-Predator Behaviour in the European Green Crab Carcinus maenas
Source: Animals (Basel). 2026 Mar 6;16(5):828. doi: 10.3390/ani16050828 (PMC12984149; doi:10.3390/ani16050828)
Supplement: Supplementary file 1 [file animals-16-00828-s001.zip › animals-4103492-supplementary.pdf]

Supplementary

Supplementary Figure S1

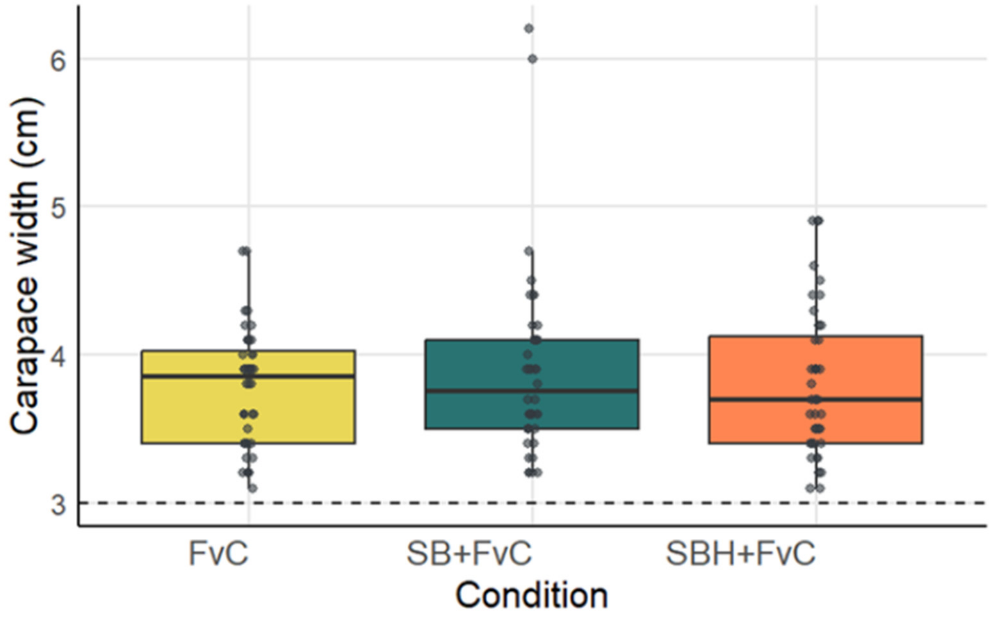

Supplementary Figure S1. Carapace width (cm) of individuals across three different treatment conditions (n=40 per treatment). Boxes represent the interquartile range (IQR) with the median indicated by the horizontal line; whiskers extend to 1.5 × IQR. Individual data points are overlaid using jitter to illustrate distribution. A dashed horizontal line marks a reference threshold of 3 cm for minimum size requirement._Experimental groups tested were FvC (Food vs Control), SB+FvC (Sea Bream + Food vs Control), and SBH+FvC (Sea Bream(Habituated + Food vs Control).

Supplementary Figure S2

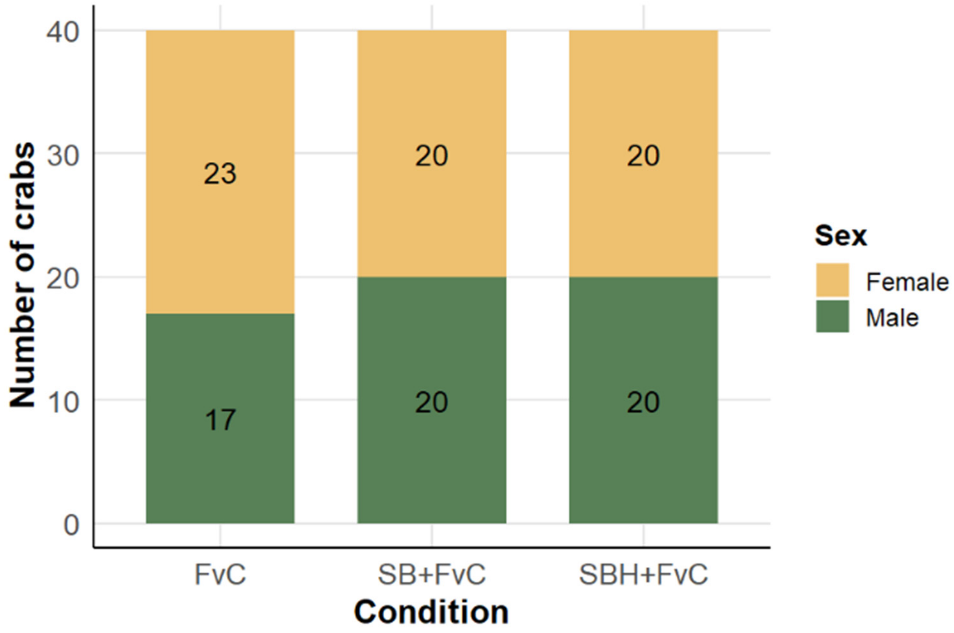

Supplementary Figure S2. Distribution of the number of male (green) and female (yellow) individuals within each treatment group (n=40 per treatment). Counts are displayed inside each bar segment to indicate sample sizes per sex. Differences in sex ratios among treatment groups were assessed using a chi-square (χ^2) test; statistical results are reported in Supplementary Analysis 2. Experimental groups tested were FvC (Food vs Control), SB+FvC (Sea Bream + Food vs Control), and SBH+FvC (Sea Bream(Habituated + Food vs Control).

Supplementary Figure S3

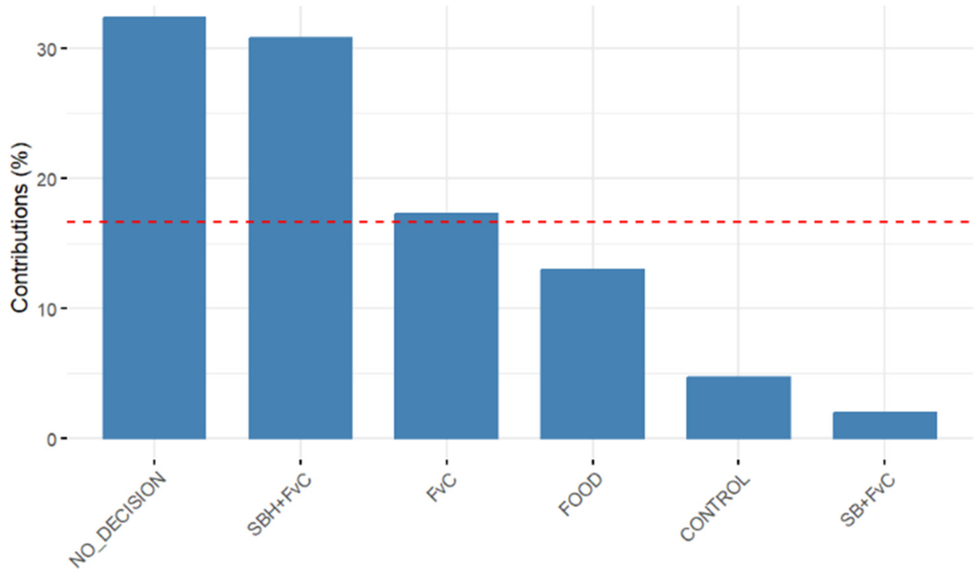

Supplementary Figure S3. Variable contributions to Dimension 1 from Multiple Correspondence Analysis (MCA) based on multinomial model input. Experimental groups tested were FvC (Food vs Control), SB+FvC (Sea Bream + Food vs Control), and SBH+FvC (Sea Bream(Habituated + Food vs Control). Bar plot showing the percentage contribution of each category to the construction of Dimension 1, calculated from the MCA performed on categorical variables used in the multinomial logistic regression model. Dimension 1 accounts for 36.9% of the total variance. The strongest contributors were no decision and SBH+FvC, indicating these variables most strongly differentiate responses along this axis. The red dashed line denotes the average expected contribution if all variables contributed equally.

Supplementary Figure S4

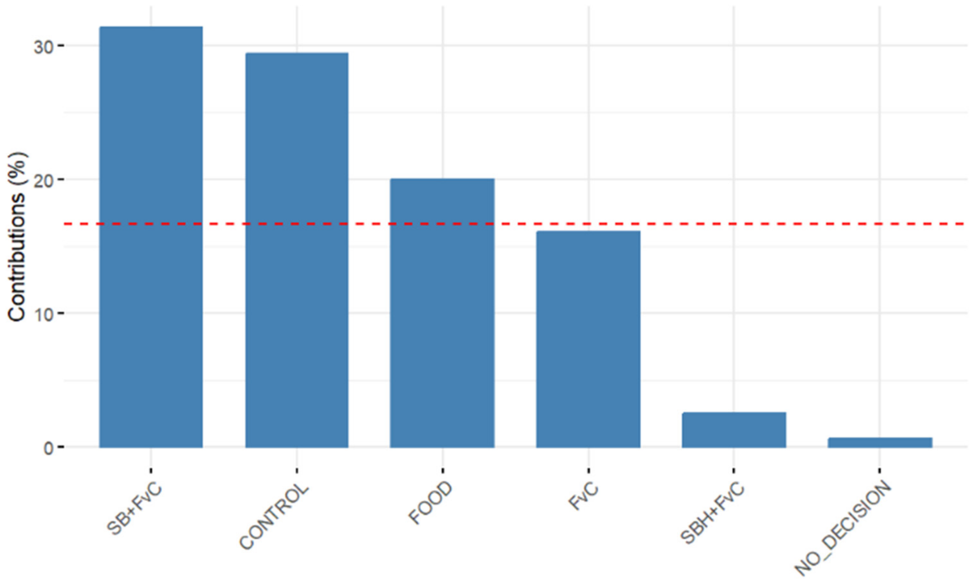

Supplementary Figure S4. Variable contributions to Dimension 2 from Multiple Correspondence Analysis (MCA) based on multinomial model input. Bar plot illustrating the contribution of each variable category to the construction of Dimension 2, derived from MCA performed on the same categorical data used in the multinomial logistic regression model. Dimension 2 explains 29.4% of the total variance. Experimental decisions were for Food, Control, FvC (Food vs Control), SB+FvC (Sea Bream + Food vs Control), and SBH+FvC (Sea Bream(Habituated + Food vs Control). SB+FvC and CONTROL were the strongest contributors to this dimension, suggesting that variation along this axis is driven largely by differences between these two treatment conditions. The red dashed line indicates the expected average contribution.

Supplementary Figure S5

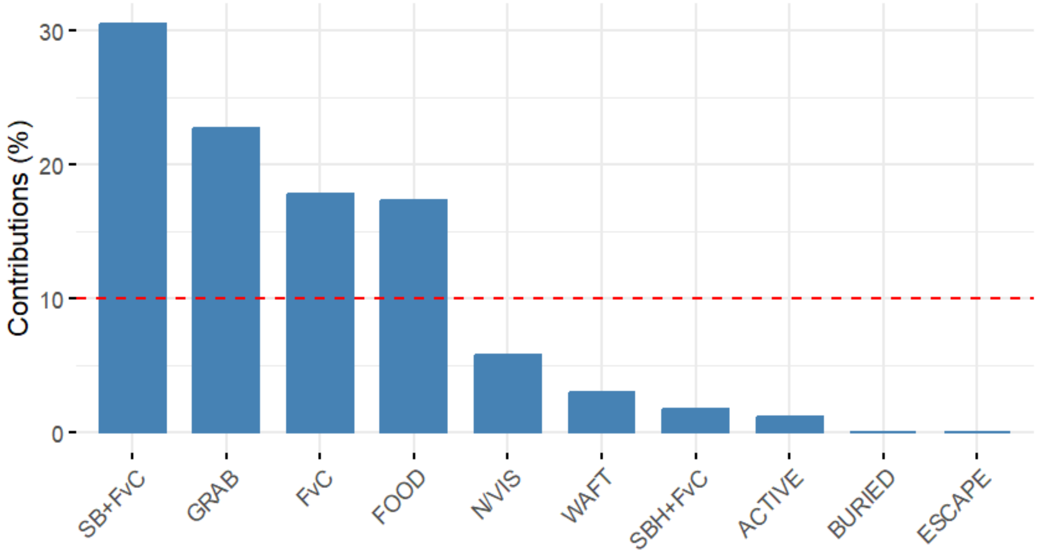

Supplementary Figure S5. Variable contributions to Dimension 1 from Multiple Correspondence Analysis (MCA) based on multinomial model input. Bar plot showing the percentage contribution of each category to the construction of Dimension 1, calculated from the MCA performed on categorical variables used in the multinomial logistic regression model. Dimension 1 accounts for 22.3% of the total variance. Experimental decisions were for Food, Control, FvC (Food vs Control), SB+FvC (Sea Bream + Food vs Control), and SBH+FvC (Sea Bream(Habituated + Food vs Control), N/VIS (No visible behavioural response), Grab, Waft, Active, Buried. The strongest contributors were grabbing and SB+FvC, indicating these variables most strongly differentiate responses along this axis. The red dashed line denotes the average expected contribution if all variables contributed equally. (Dim1 = 22.3%, Dim2 = 19.1%), which together explain 41.4%

Supplementary Figure S6

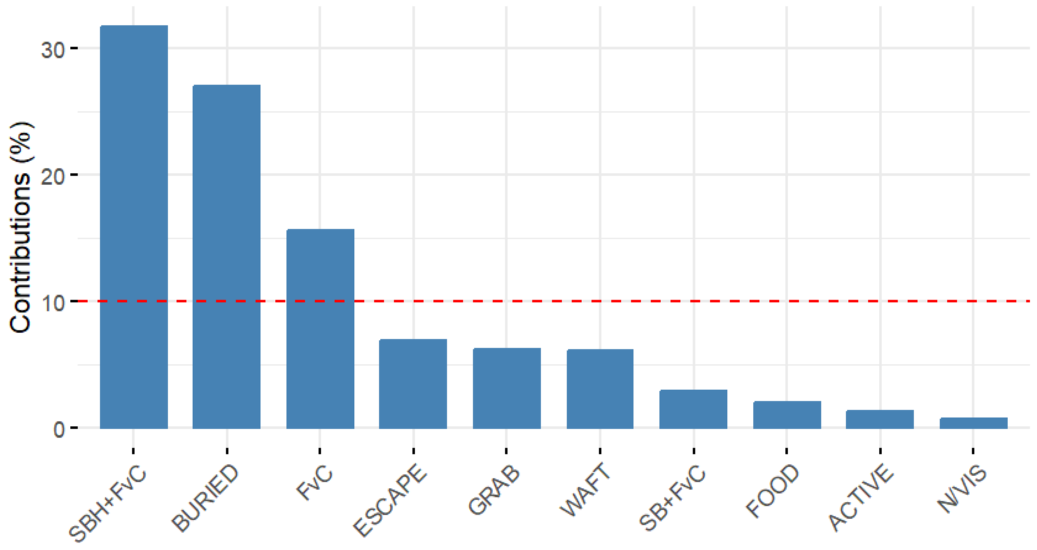

Supplementary Figure S6. Variable contributions to Dimension 2 from Multiple Correspondence Analysis (MCA) based on multinomial model input. Bar plot showing the percentage contribution of each category to the construction of Dimension 1, calculated from the MCA performed on categorical variables used in the multinomial logistic regression model. Experimental decisions were for Food, Control, FvC (Food vs Control), SB+FvC (Sea Bream + Food vs Control), and SBH+FvC (Sea Bream(Habituated + Food vs Control), N/VIS (No visible behavioural response), Grab, Waft, Active, Buried. Dimension 2 accounts for 19.1% of the total variance. The strongest contributors were burying behaviour and SBH+FvC, indicating these variables most strongly differentiate responses along this axis. The red dashed line denotes the average expected contribution if all variables contributed equally.

Supplementary Analysis 1: Analysis of crab carapace width compared to condition group

Normality test: Shapiro-Wilk normality test: W = 0.94848, p-value = 0.06725

A one-way ANOVA revealed no significant effect of treatment condition on carapace width ($F(2, 117) = 0.61$, $p = 0.546$).

Supplementary Table S1. Tukey HSD post hoc comparisons of carapace width between treatment groups following one-way ANOVA comparing carapace width (cm) across three treatment conditions. The table reports pairwise mean differences (diff), 95% confidence intervals (lower and upper bounds), and adjusted p-values for multiple comparisons. No significant differences were detected between any groups ($p > 0.05$).

Comparison	diff	Lwr	upr	p. adjusted
SB+FvC-FvC	**0.120**	**-0.162**	**0.402**	**0.571**
SBH+FvC-FvC	**0.105**	**-0.177**	**0.387**	**0.651**
SBH+FvC-SB+FvC	**-0.015**	**-0.297**	**0.267**	**0.991**

Supplementary Analysis 2: Analysis of crab sex distribution compared to condition group

Normality test: Shapiro-Wilk normality test: W = 0.86169, p-value = 1.082e-05

A chi-square test of independence revealed no significant association between sex and treatment condition ($\chi^2(2) = 0.60$, $p = 0.740$).

Supplementary Analysis 3: Multinomial Logistic Regression Model Summary

A likelihood ratio test comparing the intercept-only model (residual deviance = 263.52, df = 238) to the full model including treatment as a predictor (residual deviance = 232.89, df = 234) showed a significant improvement in model fit ($\chi^2 = 30.62$, df = 4, p = 3.65×10^{-6}), indicating that treatment significantly influences crab decision-making.

Supplementary Table S2. Multinomial Logistic Regression Coefficients for Decision Making by Treatment Condition. Parameter estimates of coefficients and standard errors from the multinomial logistic regression model predicting crab decision outcomes (Food choice, No decision) based on treatment conditions. The intercept corresponds to the log-odds of each decision category for crabs in the control group (reference level). Coefficients for the SB+FvC and SBH+FvC groups represent the change in log-odds of selecting the respective decision relative to the control. Positive coefficients indicate increased likelihood compared to control, while negative coefficients indicate decreased likelihood.

**Outcome**	**Predictor**	**Coefficient (β)**	**Std.Error**	**z-value**	**p. value**
Food	(Intercept)	**0.526**	**0.350**	**1.50**	**0.13**
Food	SB+FvC	**-0.932**	**0.511**	**-1.82**	**0.068**
Food	SBH+FvC	**-0.526**	**0.639**	**-0.82**	**0.41**
No_Decision	(Intercept)	**-0.956**	**0.526**	**-1.82**	**0.069**
No Decision	SB+FvC	**0.368**	**0.658**	**0.560**	**0.580**
No Decision	SBH+FvC	**2.268**	**0.677**	**3.35**	**0.008**

Supplementary Analysis 4: Linear Regression Model Summary

Supplementary Table S3 Linear regression model summary table examining the effect of treatment condition on time to locate the cue in shore crabs. The model included three treatment groups: CvF (Control vs Food; reference level), SB+FvC (Seabream odour + Food vs Control), and SBH+FvC (Habituated Seabream odour + Food vs Control). Reported are the model estimates, standard errors, t values, and associated p-values. Model diagnostics are as follows: residual standard error = 61.55 (df = 76); F-statistic = 2.595 (on 2 and 76 degrees of freedom); $R^2 = 0.06392$; adjusted $R^2 = 0.03929$; model p-value = 0.08125.

(Intercept)	59.857	10.404	5.753	1.74e-07
SB+FvC	34.510	15.314	2.253	0.0271
SBH+FvC	9.857	19.464	0.506	0.6140

Supplementary Table S4 Linear model summary table of linear regression model examining the effect of treatment condition initial reaction time (IR) in shore crabs. The model included three treatment groups: CvF (Control vs Food; reference level), SB+FvC (Seabream odour + Food vs Control), and SBH+FvC (Habituated Seabream odour + Food vs Control). Reported are the model estimates, standard errors, t values, and associated p-values. Model diagnostics are as follows: residual standard error = 6.381 (df = 76); F-statistic = 1.781 (on 2 and 76 degrees of freedom); $R^2 = 0.04476$; adjusted $R^2 = 0.01962$; model p-value = 0.1755.

**Term**	**Estimate.**	**Std.Error**	**t value**	**p-value**
(Intercept)	1.771	**1.079**	**1.642**	**0.105**
SB+FvC	2.995	**1.588**	**1.887**	**0.063**
SBH+FvC	1.300	**2.018**	**0.644**	**0.521**

Supplementary Analysis 5: Generalised Least Squares

Initial linear regression model: lm (Time to locate ~ Treatment* Sex* Size)

Minimal adequate model: gls (Time to locate ~ 1, weights = varIdent (form = ~ 1), method = "REML")

Outcome:

The generalized least squares (GLS) intercept-only model estimated a baseline average time to locate the cue of 74.71 seconds (standard error = 7.07, $t = 10.57$, $p < 0.001$), representing the overall mean time across all observations without inclusion of predictor variables. Residual standard error was 62.80 with 78 degrees of freedom.

Supplementary Analysis 6: Multinomial Logistic Regression Model Summary

A likelihood ratio test comparing the intercept-only model (residual deviance = 323.98, df = 595) to the full model including condition as a predictor (residual deviance = 271.56, df = 585) showed a significant improvement in model fit ($\chi^2 = 52.42$, df = 10, $p = 9.55 \times 10^{-8}$), indicating that treatment condition significantly influences crab reaction. This multinomial logistic regression model allowed us to estimate the probabilities of each behavioural reaction (ACTIVE, BURIED, ESCAPE, GRAB, N/VIS, WAFT) across the different treatment conditions. Post hoc comparisons using estimated marginal means (emmeans) and Tukey-adjusted pairwise contrasts revealed which specific treatment conditions differed in their effects on each reaction category, providing a detailed understanding of how treatments influenced crab behaviour at the level of individual response types. Estimated marginal means (emmeans) were used to compute predicted probabilities for each reaction within each treatment condition, allowing for interpretable pairwise comparisons between treatments with Tukey-adjusted p-values, rather than relying solely on log-odds coefficients from the model summary.

Supplementary Table S5. Pairwise Comparisons of Crab Behavioral Reactions by Treatment Condition. Predicted probabilities of each crab behavioral reaction (ACTIVE, BURIED, ESCAPE, GRAB, N/VIS, WAFT) were estimated from the multinomial logistic regression model using emmeans. Pairwise contrasts between treatment conditions (FvC, SB+FvC, SBH+FvC) were computed with Tukey-adjusted p-values to identify significant differences in reaction probabilities. Positive contrast estimates indicate that the first treatment in the comparison increases the likelihood of the reaction relative to the second treatment, while negative estimates indicate a decreased likelihood. This approach allows for direct and interpretable comparisons of treatment effects on crab behavior across all reaction categories.

Reaction	Contrast	Estimate	Std.Error	Df	t-ratio	p-value
ACTIVE	FvC - SB+FvC	0.025	0.0247	15	1.013	0.5805
	FvC - SBH+FvC	0.025	0.0247	15	1.013	0.5805
s	SB+FvC - SBH+FvC	0.0000009	0.000157	15	0.006	1.0000
BURIED	FvC - SB+FvC	-0.10	0.0731	15	-1.368	0.3817
	FvC - SBH+FvC	-0.50	0.0886	15	-5.646	0.0001
	SB+FvC - SBH+FvC	-0.40	0.0986	15	-4.058	0.0028
ESCAPE	FvC - SB+FvC	-0.025	0.0247	15	-1.013	0.5804
	FvC - SBH+FvC	-0.10	0.0474	15	-2.108	0.1217
	SB+FvC - SBH+FvC	-0.075	0.0535	15	-1.403	0.3645
GRAB	FvC - SB+FvC	0.325	0.103	15	3.165	0.0166
	FvC - SBH+FvC	0.375	0.099	15	3.789	0.0048
	SB+FvC - SBH+FvC	0.050	0.0893	15	0.560	0.8429
N/VIS	FvC - SB+FvC	-0.075	0.0416	15	-1.801	0.2029
	FvC - SBH+FvC	0.000	0.000	15	0.013	0.9999
	SB+FvC - SBH+FvC	0.075	0.0416	15	1.801	0.2029
WAFT	FvC - SB+FvC	-0.15	0.109	15	-1.373	0.3792
	FvC - SBH+FvC	0.20	0.0942	15	2.123	0.1186
	SB+FvC - SBH+FvC	0.35	0.0971	15	3.603	0.0069
